# Age and gender-specific flow-mediated dilation reference values and predictive factors for Chinese children and adolescents

**DOI:** 10.1186/s12887-025-05974-1

**Published:** 2025-08-07

**Authors:** Enoch C. So, Kate C. Chan, Chun T. Au, Ping Chook, Magnum K. Yu, Hung K. So, Michael H. Chan, Kam S. Woo, David S. Celermajer, Albert Martin Li

**Affiliations:** 1 Department of Paediatrics, Prince of Wales Hospital, The Chinese University of Hong Kong, Shatin, Hong Kong; 2 Laboratory for Paediatric Respiratory Research, Li Ka Shing Institute of Health Sciences, Faculty of Medicine, The Chinese University of Hong Kong, Shatin, Hong Kong; 3 Hong Kong Hub of Paediatric Excellence, The Chinese University of Hong Kong, Shatin, Hong Kong; 4 Department of Medicine and Therapeutics, Prince of Wales Hospital, Shatin, Hong Kong; 5 Department of Paediatrics and Adolescent Medicine, Queen Mary Hospital, The University of Hong Kong, Pokfulam, Hong Kong; 6 Department of Chemical Pathology, Prince of Wales Hospital, The Chinese University of Hong Kong, Shatin, Hong Kong; 7 School of Life Sciences, The Chinese University of Hong Kong, Shatin, Hong Kong; 8 Department of Cardiology, Royal Prince Alfred Hospital, Camperdown, NSW Australia

**Keywords:** Endothelial function, Flow-mediated dilatation, Reference values, Normative values, Paediatrics, Children

## Abstract

**Background:**

We aim to establish normative reference values of brachial artery flow-mediated dilation (FMD) by age and gender in children and adolescents, and to identify predictors of FMD in this population.

**Methods:**

A representative sample of 1498 healthy children and adolescents aged 8 to 17 years was recruited. Subjects underwent sonographic brachial artery assessment and blood sampling. Smoothed gender-specific FMD percentile curves were constructed using the Lambda-Mu-Sigma (LMS) method. Predictive factors of FMD were identified using linear regression analysis.

**Results:**

Mean FMD among children and adolescents in the community setting was 8.57 ± 0.90%. Smoothed gender-specific FMD in centiles were constructed as a reference benchmark. Regression analysis after adjustment for age, gender, body mass index (BMI) z-score, and baseline artery diameter, when applicable, demonstrated that FMD is positively correlated with age (β = 0.142, *p* < 0.001, 95% CI [0.081–0.203]) and high density lipoprotein (HDL) (β = 0.103, *p* = 0.001, 95% CI [0.041–0.165]), while negatively correlated with baseline artery diameter (β = -0.117, *p* = 0.001, 95% CI [-0.189 – -0.046]), diastolic blood pressure (DBP) (β = -0.053, *p* = 0.047, 95% CI [-0.105 – -0.001]), glucose (β = -0.091, *p* = 0.004, 95% CI [-0.153 – -0.030]) and triglyceride (TG) (β = -0.138, *p* < 0.001, 95% CI [-0.198 – -0.078]). Multivariate regression analysis showed that age, baseline artery diameter, DBP, glucose and TG were independent predictors of FMD.

**Conclusions:**

Normative reference values for FMD were constructed with predictive factors identified for children and adolescents.

**Supplementary Information:**

The online version contains supplementary material available at 10.1186/s12887-025-05974-1.

## Introduction

The vascular endothelial cell layer is functionally important in maintaining vascular homeostasis via tight regulation of fluid, nutrient and metabolite balance. Interruption in the layer results in the loss of vascular integrity with a pro-thrombotic state due to the up-regulation of adhesion molecules and cytokines [1]. Diseased endothelial layer silently progresses to clinically significant arterial diseases such as atherosclerosis and aneurysms in adulthood [2]. Therefore, early recognition of endothelial dysfunction and its associated modifiable risk factors, even in childhood age, is essential to halt further progression of endothelial dysfunction.

The concept of early vascular aging (EVA) and endothelial function is central to the vascular assessment in youth, with expert consensus highlighting flow-mediated dilation (FMD) as a key measure [3]. In brief, endothelial function can be assessed sonographically by measuring the brachial artery diameter both at rest and immediately following a short period of limb ischemia. In arteries lined by healthy endothelium, increased blood flow is observed post limb ischemia due to vasodilatation induced by endothelium-derived relaxing factors. The proportional increase in luminal diameter is known as flow-mediated dilation (FMD) [4]. Thus brachial artery FMD can be viewed as a surrogate marker of systemic endothelial function. In children, impaired FMD has been documented in diabetes, obesity, sleep-disordered breathing, dyslipidaemia, and Henoch-Schonlein purpura [5–8].

The clinical use of FMD depends on the availability of robust pediatric reference values, which remain limited in the published literature. Recent efforts, such as Hopkins et al. [9] and Torigoe et al. [10], have begun to address this gap. However, further research is needed to establish population-specific ranges and to identify predictive risk factors for endothelial dysfunction in pediatric populations.

Our team previously published a brief report of flow-mediated dilation results [11]. In this study, we sought to build upon that work by analyzing the complete data set, further assessing the characteristics of the normative reference values for children and adolescents, as well as identifying predictive risk factors within this population.

## Methods

Healthy Chinese children and adolescents, aged 8–17 years old, were recruited from the community by random selection of primary and secondary schools in Hong Kong. Subjects were recruited from all four geographic regions in Hong Kong. The number of students recruited from each region was proportional to the distribution of the paediatric population within that region. Sampling classes within the selected school were randomly selected. All students within the sampling class were invited to participate.

Subjects suffering from acute or chronic illness (for example, diabetes mellitus, kidney disease, dyslipidaemia, congenital or acquired heart disease, lupus erythematosus), on drug treatment that might affect blood pressure (BP) levels (non-steroidal anti-inflammatory drugs, corticosteroids, bronchodilators) or had a recent infection within the past 4 weeks were excluded.

This study was approved by the Joint Chinese University of Hong Kong– New Territories East Cluster Clinical Research Ethics Committee (CREC-2012.456). Written informed consent was obtained from the parents of each participant. The study was conducted in accordance with the Declaration of Helsinki.

### Anthropometric measurements, physical activity, and pubertal status assessment

Body mass index (BMI) was derived from weight and height measurements obtained on the day of sonographic assessment, and converted to age- and sex-appropriate z-scores using a local reference [12]. Standing height without shoes was measured with a Harpenden stadiometer (Holtain) to the nearest 0.1 cm, and body weight (in kilograms) with the lightest clothing was measured with an electronic weighing scale (Tanita BF-522, Japan) to the nearest 0.1 kg. Waist circumference was assessed midway between the lowest rib and the superior border of the iliac crest whereas the hip circumference was measured at the maximal protrusion of the buttocks [13, 14]. Self-assessment of physical activity and Tanner’s pubertal stage [15] were collected.

### Blood pressure and brachial artery flow-mediated dilation assessment

Blood pressure (BP) was measured using an Accutorr Plus monitor, an oscillometric device validated with a mercury sphygmomanometer in children [16] after resting for 10 min. Readings were taken at 5-minute intervals on the non-dominant arm at the heart level with an appropriate-sized cuff. The average of two readings was used for analysis.

The FMD measurement was carried out in a quiet, temperature-controlled room. All subjects abstained from food, including caffeine for at least 6 h before testing. Strenuous physical activity were avoided for 24 h before assessment. The diameter of the brachial artery was measured on B-mode ultrasound images (i) at rest and (ii) in response to reactive hyperaemia, which was induced by inflation of a BP cuff placed around the lower arm to a pressure of 220mmHg for 4–5 min, followed by rapid deflation. Measurements were taken using a linear array transducer (L10-5 median frequency, 7.5 MHz) and the SonoSite MicroMAX ultrasound system. To minimize variability, all measurements were taken at the end-diastole identified by the R wave on the electrocardiogram, and the average of three measurements along the vessel was taken. Measurements were performed by an experienced echocardiographer and data was recorded for subsequent analysis. This methodology was demonstrated to be reliable, valid, and children-friendly [17–19].

### Cardiovascular risk factors assessment

A voluntary blood sample was collected for analysis of fasting glucose and lipid profile analysis following the FMD assessment. Plasma glucose was measured using the hexokinase method on a C8000 Cobas 702 Clinical Chemistry Analyzer (Roche Diagnostics Corp., Indianapolis, IN, USA). The inter-assay coefficient of variation was ≤ 3% at all concentrations above 1.6 mmol/L. Lipid profiles were analyzed using standard enzymatic assays on the same analyzer, with an inter-assay coefficient of variation ≤ 3% at all concentrations above 0.5 mmol/L.

### Statistical analysis

Student’s *t*-test and chi-square test were used for group comparisons for numeric and categorical data respectively. Adjusted FMD was calculated with covariate controlled for baseline artery diameter, allowing FMD to be scaled for changes in artery diameter. Effects of demographic, anthropometric variables, and cardiovascular risk factors on FMD were examined using linear regression analysis. The level of significance was defined as 0.05. Statistical software (SPSS; Chicago, IL) was used for all analyses.

Percentile curves for FMD were constructed using the Lambda-Mu-Sigma (LMS) method [20]. The LMS method estimates the measurement centiles in terms of three age- and gender-specific cubic spline curves: the L curve (Box-Cox power to transform the data that follow a normal distribution), the M curve (median), and the S curve (coefficient of variation). In brief, if Y(t) denotes independent positive data at t age (years), the distribution of Y(t) can be summarised by a normally distributed SD score (Z) as follows:

$$Z=\frac{\left[Y\left(t\right)/M\left(t\right)^{L\left(t\right)}-1\right]}{L\left(t\right)S\left(t\right)}$$

Once the L(t), M(t) and S(t) have been estimated for each age t, the 100α^th^ centile at t could be derived from

$$C_{100\alpha}\left(t\right)=M\left(t\right)\left[1+L\left(t\right)S\left(t\right)Z_\alpha\right]^{1/L\left(t\right)}$$

Zα as α centile of the normal distribution (e.g., for the 97th centile, α = 0.75 and Zα = 1.88).

Repeatability of FMD measurements was assessed on a subgroup of randomly selected subjects using paired measurements performed at baseline and within four weeks later using Bland-Altman plot [21] and paired-sample t-test analysis.

### Sample size calculation

We assumed FMD was normally distributed among each age and gender, and age- and the gender-specific sample size was calculated in terms of the standard deviation of the 100th centile (c100α, alpha = 0.05 for 95th percentile) using the sample size planning formula [20]. To define the age and gender-specific mean and standard deviation (SD) of FMD, pilot data were collected from 93 to 81 healthy primary (aged 8–12 years) and secondary school (aged 12–17 years) students, respectively. From the pilot study, SD for the primary and secondary school subjects were 0.84 and 1.16 respectively. To obtain an extreme centile, i.e. the 97th centile, with an error of ± 2.5%, the estimated number of primary and secondary school students was 45 and 85 for each year of age, resulting in a total of 1380 subjects.

## Results

Six primary schools and 14 secondary schools were recruited with three classes from each school randomly selected to join the study. 194 students declined to participate and eleven students were excluded due to chronic illness. In total, 1498 students were recruited and analysed.

Male subjects were taller, heavier, and with greater waist and hip circumference than females. More female subjects attained puberty in the sample, reflecting their earlier onset of puberty. There was no significant difference in BMI or BMI z-score between gender. Systolic and diastolic blood pressure were statistically significantly higher in male than female subjects, while heart rate was lower in male than female subjects (Table 1).

**Table 1:** Descriptive statistics of study sample by gender

		Total	Male	Female	*p*
*n*		1498	759	739	
Age, yr		13.42 ± 2.71	13.40 ± 2.71	13.43 ± 2.71	0.822
Weight, kg		46.05 ± 13.88	47.72 ± 15.17	44.34 ± 12.20	< 0.001^a^
Height, cm		154.25 ± 14.06	156.51 ± 15.57	151.93 ± 11.88	< 0.001 ^a^
BMI, kg/m2		18.90 ± 3.42	18.95 ± 3.43	18.85 ± 3.41	0.586
BMI-z score		0.149 ± 0.943	0.155 ± 0.915	0.142 ± 0.974	0.779
Waist to Hip ratio		0.801 ± 0.629	0.812 ± 0.566	0.790 ± 0.669	< 0.001 ^a^
Tanner staging					0.010*
Stage 1–3		768 (51.3%)	414 (54.5%)	354 (47.9%)	
Stage 4–5		730 (48.7%)	345 (45.5%)	385 (52.1%)	
SBP, mmHg		109.06 ± 10.75	111.22 ± 11.30	106.83 ± 9.66	< 0.001 ^a^
DBP, mmHg		63.59 ± 7.82	64.61 ± 8.11	62.55 ± 7.37	< 0.001 ^a^
Heart rate, bpm		80.98 ± 13.11	79.83 ± 13.11	82.01 ± 13.04	0.005 ^a^
FMD, %		8.57 ± 0.90	8.53 ± 0.99	8.61 ± 0.78	0.081
Adjusted FMD, %		8.57 (8.52–8.61)	8.54 (8.47–8.60)	8.60 (8.53–8.67)	0.203
Baseline artery diameter, mm		2.58 ± 0.39	2.74 ± 0.40	2.41 ± 0.29	< 0.001 ^a^

Data presented as Mean (95% confidence interval of mean) for adjusted FMD, Mean ± SD for all other continuous variables, or frequency (percentage of subgroup) for categorical variable tanner staging

*Adjusted FMD* FMD adjusted by baseline artery diameter, *BMI* Body mass index, *DBP* Diastolic blood pressure, *FMD* Flow-mediated dilation, *n* Sample size, *SBP* Systolic blood pressure

^a^Denotes statistical significance at 0.05

The mean FMD of this group of healthy children and adolescents in the community setting was 8.57 ± 0.90%. Overall no statistical significance was observed for FMD by gender (males 8.53 ± 0.99% vs. females 8.61 ± 0.78%, *p* = 0.081) (Table 1). Subgroup analysis revealed that significance was attained in male teenagers aged 12 years or above with a lower FMD value compared to females (males 8.55 ± 1.05% vs. females 8.69 ± 0.76%, *p* = 0.015). However, the teenage gender difference appeared to be mediated by baseline artery diameter, with the difference attenuated following adjustment of arterial diameter on regression analysis (*p* = 0.236). No significant difference between gender was observed in children aged 11 years or less (males 8.48 ± 0.82% vs. females 8.43 ± 0.82%, *p* = 0.481).

**Fig. 1:**
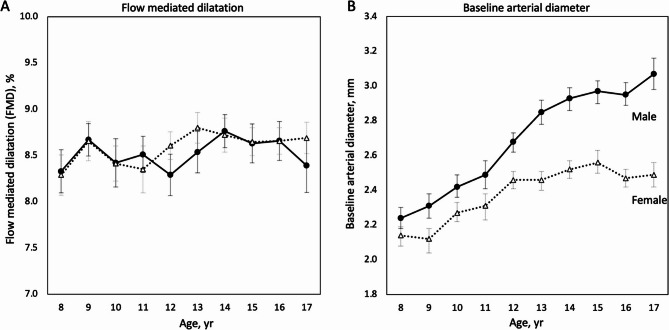
Flow mediated dilatation (FMD) **A** and baseline arterial diameter **B** by age and gender. Solid line with solid black circular makers represent male; Dashed line with unfilled triangular markers represent female. Error bars denote 95% confidence interval for respective gender. Total number of subjects was 1498

**Fig. 2:**
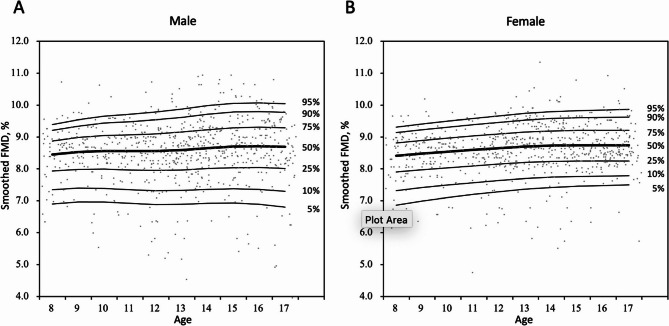
Smoothed FMD (%) in centiles by LMS method in male **A** and female **B**. Grey circular markers represent flow mediated dilatation of individual patients in sample. Total number of subjects was 1498. FMD, Flow mediated dilation

The absolute difference in FMD by age was small but a trend was notable. Excluding the potential outlier subgroup at age nine, FMD was lowest at eight years of age (8.31 ± 0.85%), initially trended upwards gradually but accelerated at age 13, peaked at age 14 at 8.74 ± 0.90%, before declining at age 17 (8.54 ± 1.02%). FMD for females peaked one year earlier at age 13 (8.80 ± 0.82%) compared to males at age 14 (8.76 ± 0.90%). After their respective peak in adolescence, FMD declined more steeply for males (8.39 ± 1.23%), whereas FMD for females plateaued and fluctuated tightly between 8.65 and 8.72% (Fig. 1: Panel A) (Supplementary Tables 1–3). Smoothed FMD curves reported in centiles by gender were constructed for future benchmarking (Fig. 2) (Supplementary Table 4).

Male subjects exhibited a greater mean baseline brachial artery diameter compared to females in our population (males 2.74 ± 0.40 mm vs. females 2.41 ± 0.29 mm, *p* < 0.001). The difference was statistically significant in all age groups above nine years old in subgroup analysis. Baseline artery diameter increased with age for males with a peak attained at age 17, whereas for females it peaked at age 15 before gently declining (Fig. 2: Panel B).

**Fig. 3:**
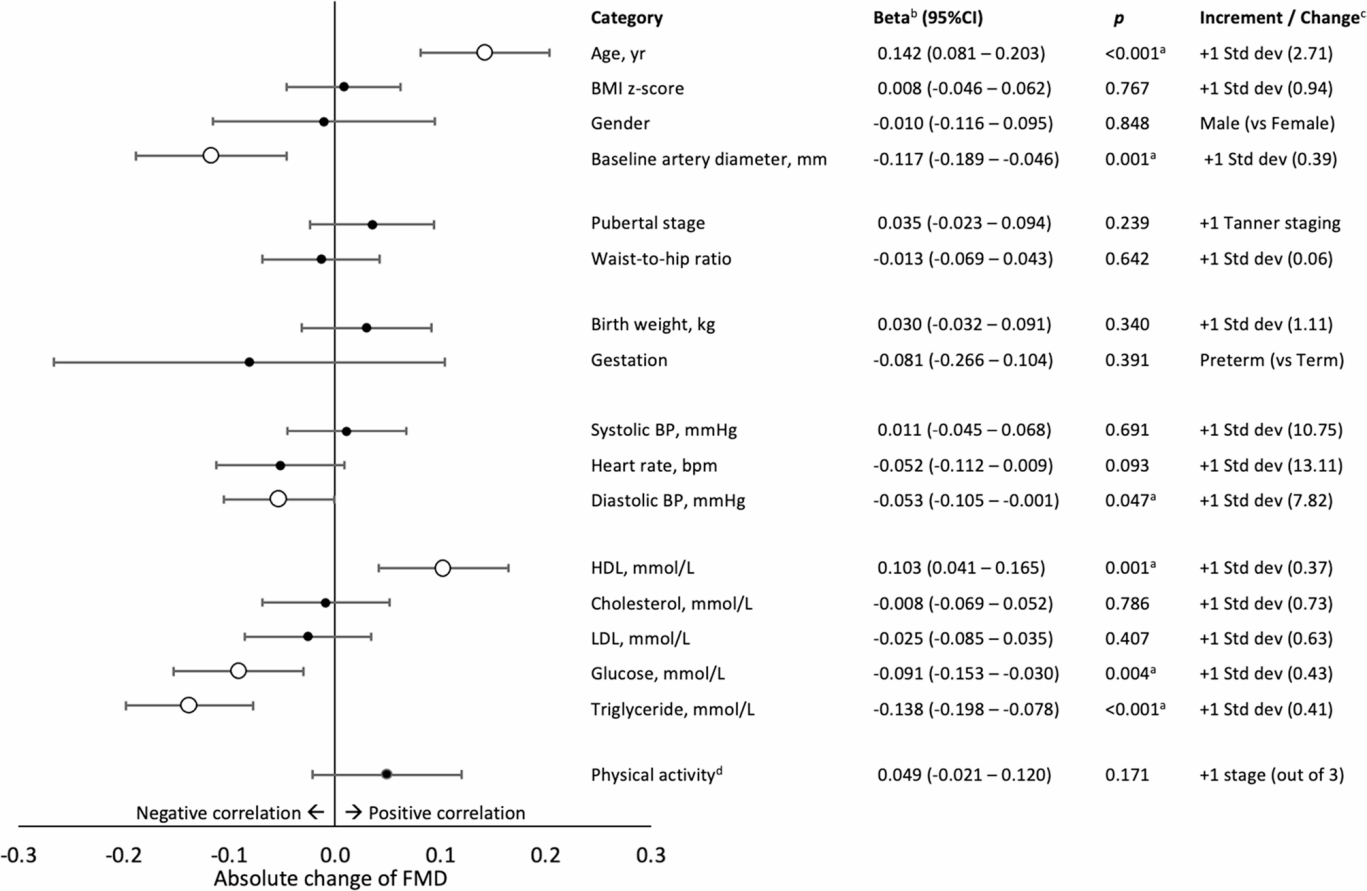
Absolute change on FMD (%) for each incremental change on variables. Each variable is adjusted for age, gender, BMI-z score and baseline artery diameter when applicable. Unfilled large circular marker indicates statistical significance with *p* < 0.05. Total number of subjects was 1498. Linear regression analysis was applied with each variable adjusted for age, gender, BMI-z score and baseline artery diameter when applicable. BP: Blood pressure; BMI z-score: Body mass index z-score; FMD: Flow mediated dilation; Std dev: Standard deviation. ^a^Denotes statistical significance at 0.05. ^b^Beta for all categorical variables including gender, pubertal stage, gestation and physical activity are reported as unstandardised beta with displayed change as increment. Beta for all continuous variables are reported as standardised beta with respective standard deviation as increment. ^c^Increment/Change for all categorical variables are reported as displayed, and for all continuous variables are reported as one additional standard deviation with actual standard deviation displayed within parenthesis. ^d^Physical activity is graded in three stages– mild, moderate and vigorous

Regression analysis with adjustments for known confounding factors including age, gender, BMI z-score, and baseline artery diameter, when applicable, was performed to assess the independent effects of variables on FMD (Fig. 3) (Supplementary Table 5). Baseline artery diameter demonstrated a negative correlation with FMD (β = −0.117, *p* = 0.001). Conversely, age demonstrated a positive correlation with FMD (β = 0.142, *p* < 0.001). Subgroup analysis confirmed the positive correlation of age was present in both male (β = 0.126, *p* = 0.009) and female (β = 0.164, *p* < 0.001). No significant correlations with FMD were noted for gender (B = −0.01, *p* = 0.848), BMI z-score (β = 0.008, *p* = 0.767), waist-to-hip ratio (β = −0.013, *p* = 0.642), pubertal stage (B = 0.035, *p* = 0.239), gestation (B = −0.081, *p* = 0.391) and birth weight (β = 0.030, *p* = 0.340). For blood pressure parameters, diastolic blood pressure (DBP) (β = −0.053, *p* = 0.047) showed a negative correlation with FMD. Systolic blood pressure (SBP) (β = 0.011, *p* = 0.691) and heart rate (β = −0.052, *p* = 0.093) both failed to demonstrate any significance. For blood parameters, high-density lipoprotein (HDL) (β = 0.103, *p* = 0.001) was positively correlated with FMD, whereas glucose (β = −0.091, *p* = 0.004) and triglyceride (TG) (β = −0.138, *p* < 0.001) were negatively correlated. No correlation existed for cholesterol (β = −0.008, *p* = 0.786) and low-density lipoprotein (LDL) (β = −0.025, *p* = 0.407). Physical activity also did not demonstrate correlation with FMD (B = 0.049, *p* = 0.171).

Multivariate regression analysis of aforementioned statistically significant variables confirmed that age (*p* = 0.001), baseline artery diameter (*p* = 0.049), DBP (*p* = 0.048), TG (*p* = 0.005), and glucose (*p* = 0.028) were independent predictors. HDL (*p* = 0.093) missed the level of significance (Supplementary Table 5).

Repeatability of FMD measurements assessed on a subgroup of 18 subjects demonstrated a paired sample correlation of 0.979. Repeatability coefficient was 0.217. The mean difference between the two paired measurements was 0.028 (95% CI: −0.270 to 0.825, *p* = 0.299). All except one measurement were within the statistically defined upper and lower level of agreement on Bland Altman plot. No systematic bias was noted (Supplementary Fig. 1).

## Discussion

The study, as the largest community-based representative paediatric cohort at the time of writing, provided essential FMD reference values for healthy children and adolescents.

Similarities and differences were observed between our study and published literature. Firstly, our overall mean adjusted FMD of 8.57% (95% CI [8.52–8.61]) was similar but slightly higher than the overall mean adjusted FMD of 8.44% (95% CI [6.67–10.24)) reported by Hopkins et al. [9]. Age-to-age differences and age-specific confidence intervals were smaller in our study (Supplementary Table 6). Baseline artery diameters were also smaller for all age groups by gender in our population. We postulate the sample size and representation variations may partially explain the disparity. The different methodologies employed for collecting sonographic data may also explain the differences observed. Hopkins et al. [9] measurements were defined post-procedure based on semi-automated software on sonographic recordings, whereas measurements in our study were collected from an experienced echocardiographer based on an average of three measurements.

On gender differences, our study demonstrated an overall insignificant difference between gender in FMD (males 8.53% vs. females 8.61%, *p* = 0.081). Subgroup analysis revealed that teenage females above 12 years of age attained a significantly higher FMD with our analysis showing the difference was largely mediated by baseline artery diameter. Interestingly, Hopkins et al. [9] demonstrated significant gender difference on FMD (males 7.62% vs. females 8.31%, *p* = 0.024). On closer assessment, however, the difference was also driven by the older subgroup from age 16 onwards. Both studies showcased a unique phenomenon of FMD where no major differences were observed in childhood and early teens, yet disparity exists in later teens. This hints that gender-specific differences in endothelial function are present post-puberty. Oestrogen is known to promote systemic vasodilatation and enhance microvascular reactivity, and studies have shown that post-menopausal females on hormonal replacement therapy have smaller brachial artery diameters and higher FMD [22]. Androgen appeared to have a negative effect on endothelial function [23].

With regards to age and FMD, Holder et al. [24] reported a negative correlation between age and FMD in a cohort with both paediatric and adult subjects. On the contrary, our paediatric-only cohort study showed a positive correlation between age and FMD for both gender groups even after adjustment for baseline artery diameter and BMI z-score. We hypothesize that data from Holder et al. may have a strong adulthood influence on the overall direction of correlation and thus may have concealed the trends in the paediatric subjects.

Regarding baseline artery diameter, it increased with age and was significantly larger in males compared to females (Fig. 1 Panel B). Its inverse relationship with FMD was significant even after adjusting for covariates including age, gender, and BMI z-score. This phenomenon was also well documented in the literature across all ages [24–26]. Post-ischaemic hyperaemic systolic flow has a linear relationship with the squared radius of the resting blood artery, hence the hyperaemic shear stress a smaller artery has to endure is relatively greater than a larger artery. Therefore, a higher FMD is to be expected in a smaller artery which in itself does not solely reflect the endothelial function [27]. Hence, in our study, FMD adjusted for baseline artery diameter was used to compare findings with other studies.

Concerning blood pressure’s effect on FMD, our study revealed that higher diastolic blood pressure was associated with a lower FMD in a paediatric community cohort. This is in congruence with evidence from adult studies that endothelial function is impaired for patients with elevated blood pressure [28]. It has been hypothesized that arterial wall remodelling may occur as early as the pre-pubertal stage in obese children with concurrent elevated ambulatory blood pressure [29], resulting in increased vessel wall stiffness and associated lower FMD. Our study adds to the body of evidence that even after adjustment for body size and age the association still stands.

On FMD’s relationship with lipid and glucose, previous studies showcased the detrimental effects of type 1 diabetes or dyslipidaemia on FMD [30, 31]. It is interesting to note that these effects extend to a community study as well further validating this adverse relationship. More specifically in our study for lipid profile, triglyceride and HDL displayed correlations with FMD while cholesterol does not. Interventional data on endothelial function in children is limited, but evidence supports the effects of statin on enhancing endothelial function in dyslipidaemic children [30].

Although previous studies reported physical activities to vary across age groups and impose an impact on FMD [32], our study did not establish any significant association between physical activity level and FMD. The self-reporting nature of the physical activity may not be sufficiently sensitive to detect subtle differences.

While one may argue in using a standard single reference of FMD for paediatrics due to minor differences in age and gender, we believe the uniqueness in trends and significant differences highlighted in this study may only be preserved and presented by establishing age and gender-specific endothelial function reference range.

Strengths of our study included a large representative cohort with the number of subjects recruited based on a priori sample size calculation. Described baseline differences observed between gender including weight, height, and blood pressure were similar to published references in Hong Kong [33]. There was a good balance between gender in each age group. All FMD measurements were obtained by a single investigator independent of statistical analysis. Low standard deviations were observed within subgroup analysis thus enhancing the confidence in the reported results. Repeatability analysis of FMD demonstrated good agreement between FMD readings over a four week period.

There were limitations to our study. Firstly, the subjects were conducted in mostly Chinese subjects and may have limitations in its applicability in other ethnicity. Secondly, we did not examine vasodilation independent of the endothelium with sublingual nitroglycerine due to prolonged testing time and the potential to cause discomfort to our subjects. Thirdly, our study was a cross-sectional study. Longitudinal studies on whether improvement in endothelial function during childhood would translate to decreased risk of future cardiovascular diseases would be desirable.

## Conclusions

Our study, the largest community-based representative paediatric cohort at the time of writing, provided FMD reference values for healthy children and adolescents aged 8–17 years old. Significant predictors and their effect on FMD in the paediatric cohort were exemplified. The availability of such data will allow a more meaningful assessment of cardiovascular risks in the paediatric population for future clinical and research use.

## Supplementary Information

Supplementary Material 1.

## Data Availability

The corresponding author will provide deidentified data upon request.
