# Supplementary material for: Age and gender-specific flow-mediated dilation reference values and predictive factors for Chinese children and adolescents
Source: BMC Pediatr. 2025 Aug 7;25:610. doi: 10.1186/s12887-025-05974-1 (PMC12330076; doi:10.1186/s12887-025-05974-1)
Supplement: Supplementary file 1 — Supplementary Material 1. [file 12887_2025_5974_MOESM1_ESM.docx]

**Supplementary Table 1:** Descriptive data of all subjects by age

|  |  |  | |  | FMD, % | |  | |  |
| --- | --- | --- | --- | --- | --- | --- | --- | --- | --- |
| Age, yr | *n* | Height, cm | Weight, kg | BMI, kg/m2 | Primary data | Adjusted | Baseline artery diameter, mm | SBP, mmHg | DBP, mmHg |
| 8 | 114 | 129.08 ± 5.60 | 27.27 ± 4.80 | 16.28 ± 2.06 | 8.31 ± 0.85 | 8.22 (8.02 - 8.36) | 2.19 ± 0.22 | 103.18 ± 9.70 | 60.89 ± 8.03 |
| 9 | 112 | 133.62 ± 5.58 | 27.29 ± 3.47 | 15.24 ± 1.27 | 8.66 ± 0.72 | 8.55 (8.38 - 8.73) | 2.21 ± 0.27 | 104.64 ± 9.52 | 62.63 ± 7.18 |
| 10 | 110 | 139.24 ± 5.87 | 31.74 ± 4.61 | 16.30 ± 1.58 | 8.42 ± 0.84 | 8.33 (8.18 - 8.52) | 2.35 ± 0.24 | 105.08 ± 9.46 | 62.64 ± 7.90 |
| 11 | 109 | 147.27 ± 6.82 | 36.32 ± 5.56 | 16.67 ± 1.73 | 8.43 ± 0.83 | 8.33 (8.21 - 8.55) | 2.40 ± 0.31 | 105.80 ± 9.77 | 61.96 ± 7.59 |
| 12 | 182 | 154.37 ± 6.28 | 47.26 ± 10.43 | 19.70 ± 3.53 | 8.45 ± 0.93 | 8.44 (8.31 - 8.57) | 2.57 ± 0.28 | 109.56 ± 9.91 | 62.96 ± 7.12 |
| 13 | 195 | 158.71 ± 7.04 | 47.67 ± 7.24 | 18.86 ± 2.12 | 8.67 ± 0.99 | 8.65 (8.56 - 8.81) | 2.66 ± 0.36 | 107.01 ± 9.26 | 62.10 ± 7.12 |
| 14 | 193 | 162.74 ± 7.20 | 53.24 ± 8.38 | 20.08 ± 2.78 | 8.74 ± 0.90 | 8.76 (8.66 - 8.91) | 2.73 ± 0.35 | 111.93 ± 9.49 | 63.75 ± 7.18 |
| 15 | 165 | 164.21 ± 8.60 | 55.88 ± 10.24 | 20.77 ± 3.90 | 8.64 ± 0.84 | 8.65 (8.55 - 8.83) | 2.77 ± 0.35 | 111.47 ± 10.33 | 64.07 ± 7.94 |
| 16 | 173 | 164.84 ± 8.00 | 55.34 ± 11.14 | 20.27 ± 3.09 | 8.66 ± 0.84 | 8.65 (8.56 - 8.83) | 2.71 ± 0.37 | 112.54 ± 12.67 | 66.05 ± 8.62 |
| 17 | 145 | 165.18 ± 8.07 | 57.96 ± 12.43 | 21.15 ± 3.66 | 8.54 ± 1.02 | 8.55 (8.45 - 8.74) | 2.78 ± 0.45 | 113.91 ± 10.98 | 67.49 ± 7.58 |
| Total | 1498 | 154.25 ± 14.06 | 46.05 ± 13.88 | 18.90 ± 3.42 | 8.57 ± 0.90 | 8.57 (8.52 - 8.61) | 2.58 ± 0.39 | 109.06 ± 10.75 | 63.59 ± 7.82 |

Data presented as Mean (95% confidence interval of mean) for adjusted FMD, Mean ± SD for all other continuous variables.
Adjusted, FMD adjusted by baseline artery diameter; BMI, body mass index; DBP, diastolic blood pressure; FMD, flow-mediated dilation; n: sample size; SBP, systolic blood pressure.

**Supplementary Table** **2:** Descriptive data by age in males

|  |  |  | |  | FMD, % | |  | |  |
| --- | --- | --- | --- | --- | --- | --- | --- | --- | --- |
| Age, yr | *n* | Height, cm | Weight, kg | BMI, kg/m2 | Primary data | Adjusted | Baseline artery diameter, mm | SBP, mmHg | DBP, mmHg |
| 8 | 57 | 129.35 ± 5.89 | 27.27 ± 4.91 | 16.20 ± 1.97 | 8.33 ± 0.88 | 8.23 (7.99 - 8.47) | 2.24 ± 0.23 | 103.32 ± 9.60 | 61.05 ± 8.47 |
| 9 | 56 | 134.24 ± 5.77 | 28.10 ± 3.51 | 15.55 ± 1.28 | 8.67 ± 0.65 | 8.59 (8.36 - 8.83) | 2.31 ± 0.25 | 104.77 ± 9.30 | 62.73 ± 7.40 |
| 10 | 56 | 138.50 ± 5.41 | 31.79 ± 4.80 | 16.49 ± 1.63 | 8.42 ± 0.97 | 8.38 (8.14 - 8.61) | 2.42 ± 0.25 | 104.93 ± 8.32 | 62.04 ± 6.91 |
| 11 | 57 | 145.87 ± 6.26 | 35.67 ± 5.55 | 16.68 ± 1.70 | 8.51 ± 0.74 | 8.49 (8.26 - 8.72) | 2.49 ± 0.30 | 103.88 ± 9.25 | 62.37 ± 7.95 |
| 12 | 92 | 154.30 ± 7.22 | 47.93 ± 11.76 | 19.93 ± 3.79 | 8.29 ± 1.08 | 8.32 (8.13 - 8.50) | 2.68 ± 0.28 | 111.22 ± 9.32 | 64.12 ± 7.05 |
| 13 | 99 | 160.38 ± 8.30 | 48.39 ± 8.10 | 18.72 ± 2.19 | 8.54 ± 1.12 | 8.61 (8.43 - 8.79) | 2.85 ± 0.34 | 108.66 ± 10.06 | 62.36 ± 8.20 |
| 14 | 99 | 166.47 ± 6.37 | 54.96 ± 7.48 | 19.83 ± 2.44 | 8.76 ± 0.90 | 8.86 (8.67 - 9.04) | 2.93 ± 0.32 | 115.33 ± 9.36 | 65.34 ± 7.02 |
| 15 | 86 | 168.91 ± 7.79 | 56.28 ± 8.67 | 19.68 ± 2.40 | 8.63 ± 0.98 | 8.73 (8.54 - 8.93) | 2.97 ± 0.30 | 114.67 ± 10.61 | 65.10 ± 7.60 |
| 16 | 86 | 170.16 ± 6.43 | 61.52 ± 11.92 | 21.22 ± 3.70 | 8.66 ± 0.98 | 8.76 (8.56 - 8.96) | 2.95 ± 0.31 | 118.57 ± 11.68 | 68.80 ± 9.05 |
| 17 | 71 | 170.67 ± 5.87 | 63.48 ± 13.58 | 21.77 ± 4.40 | 8.39 ± 1.23 | 8.52 (8.30 - 8.74) | 3.07 ± 0.37 | 118.25 ± 10.7 | 69.82 ± 6.76 |
| Total | 759 | 156.51 ± 15.57 | 47.72 ± 15.17 | 18.95 ± 3.43 | 8.53 ± 0.99 | 8.54 (8.47 - 8.60) | 2.74 ± 0.40 | 111.22 ± 11.30 | 64.61 ± 8.11 |

Data presented as Mean (95% confidence interval of mean) for adjusted FMD, Mean ± SD for all other continuous variables.
Adjusted, FMD adjusted by baseline artery diameter; BMI, body mass index; DBP, diastolic blood pressure; FMD, flow-mediated dilation; n: sample size; SBP, systolic blood pressure.

**Supplementary Table 3:** Descriptive data by age in females

|  |  |  | |  | FMD, % | |  | |  |
| --- | --- | --- | --- | --- | --- | --- | --- | --- | --- |
| Age, yr | *n* | Height, cm | Weight, kg | BMI, kg/m2 | Primary data | Adjusted | Baseline artery diameter, mm | SBP, mmHg | DBP, mmHg |
| 8 | 57 | 128.82 ± 5.33 | 27.27 ± 4.72 | 16.36 ± 2.16 | 8.29 ± 0.82 | 8.16 (7.91 - 8.40) | 2.14 ± 0.21 | 103.04 ± 9.88 | 60.73 ± 7.64 |
| 9 | 56 | 133.00 ± 5.36 | 26.48 ± 3.27 | 14.93 ± 1.19 | 8.65 ± 0.79 | 8.51 (8.27 - 8.76) | 2.12 ± 0.25 | 104.52 ± 9.82 | 62.54 ± 7.02 |
| 10 | 54 | 140.00 ± 6.27 | 31.68 ± 4.45 | 16.12 ± 1.51 | 8.41 ± 0.70 | 8.33 (8.08 - 8.57) | 2.27 ± 0.21 | 105.24 ± 10.60 | 63.26 ± 8.83 |
| 11 | 52 | 148.81 ± 7.13 | 37.03 ± 5.54 | 16.66 ± 1.78 | 8.35 ± 0.91 | 8.27 (8.02 - 8.51) | 2.31 ± 0.28 | 107.90 ± 9.98 | 61.52 ± 7.24 |
| 12 | 90 | 154.45 ± 5.20 | 46.58 ± 8.89 | 19.47 ± 3.25 | 8.61 ± 0.71 | 8.57 (8.39 - 8.76) | 2.46 ± 0.24 | 107.87 ± 10.25 | 61.77 ± 7.03 |
| 13 | 96 | 156.99 ± 4.93 | 46.93 ± 6.18 | 19.01 ± 2.05 | 8.80 ± 0.82 | 8.77 (8.59 - 8.95) | 2.46 ± 0.26 | 105.31 ± 8.08 | 61.82 ± 5.84 |
| 14 | 94 | 158.81 ± 5.82 | 51.43 ± 8.92 | 20.35 ± 3.09 | 8.72 ± 0.90 | 8.71 (8.53 - 8.89) | 2.52 ± 0.24 | 108.34 ± 8.27 | 62.07 ± 7.00 |
| 15 | 79 | 159.09 ± 6.20 | 55.45 ± 11.76 | 21.96 ± 4.80 | 8.65 ± 0.67 | 8.65 (8.45 - 8.84) | 2.56 ± 0.28 | 107.97 ± 8.84 | 62.94 ± 8.20 |
| 16 | 87 | 159.59 ± 5.57 | 49.23 ± 5.65 | 19.32 ± 1.94 | 8.66 ± 0.67 | 8.63 (8.44 - 8.82) | 2.47 ± 0.23 | 106.59 ± 10.68 | 63.33 ± 7.25 |
| 17 | 74 | 159.92 ± 6.18 | 52.66 ± 8.35 | 20.55 ± 2.67 | 8.69 ± 0.73 | 8.67 (8.46 - 8.87) | 2.49 ± 0.30 | 109.74 ± 9.60 | 65.26 ± 7.69 |
| Total | 739 | 151.93 ± 11.88 | 44.34 ± 12.20 | 18.85 ± 3.41 | 8.61 ± 0.78 | 8.60 (8.53 + 8.67) | 2.41 ± 0.29 | 106.83 ± 9.66 | 62.54 ± 7.37 |

Data presented as Mean (95% confidence interval of mean) for adjusted FMD, Mean ± SD for all other continuous variables.
Adjusted, FMD adjusted by baseline artery diameter; BMI, body mass index; DBP, diastolic blood pressure; FMD, flow-mediated dilation; n: sample size; SBP, systolic blood pressure.

**Supplementary Table 4**. Smoothed FMD (%) in centiles by LMS method categorised by age and gender

| Age, yr |  | Centile, % | | | | | | | | | | | | | | |
| --- | --- | --- | --- | --- | --- | --- | --- | --- | --- | --- | --- | --- | --- | --- | --- | --- |
|  |  |  |  |  | Male |  |  |  |  |  |  |  | Female |  |  |  |
|  |  | 5 | 10 | 25 | 50 | 75 | 90 | 95 |  | 5 | 10 | 25 | 50 | 75 | 90 | 95 |
| 8 |  | 6.90 | 7.35 | 7.93 | 8.45 | 8.88 | 9.21 | 9.39 |  | 6.86 | 7.32 | 7.91 | 8.41 | 8.82 | 9.14 | 9.31 |
| 9 |  | 6.96 | 7.39 | 7.99 | 8.53 | 8.98 | 9.34 | 9.54 |  | 7.00 | 7.41 | 7.98 | 8.48 | 8.90 | 9.23 | 9.41 |
| 10 |  | 6.96 | 7.39 | 7.99 | 8.56 | 9.05 | 9.44 | 9.65 |  | 7.11 | 7.50 | 8.04 | 8.54 | 8.97 | 9.31 | 9.50 |
| 11 |  | 6.92 | 7.35 | 7.97 | 8.56 | 9.07 | 9.48 | 9.72 |  | 7.20 | 7.57 | 8.10 | 8.60 | 9.03 | 9.39 | 9.58 |
| 12 |  | 6.88 | 7.32 | 7.95 | 8.56 | 9.10 | 9.54 | 9.78 |  | 7.29 | 7.64 | 8.15 | 8.65 | 9.10 | 9.46 | 9.67 |
| 13 |  | 6.89 | 7.32 | 7.97 | 8.59 | 9.16 | 9.62 | 9.88 |  | 7.36 | 7.70 | 8.20 | 8.70 | 9.15 | 9.53 | 9.74 |
| 14 |  | 6.93 | 7.36 | 8.01 | 8.65 | 9.23 | 9.71 | 9.98 |  | 7.42 | 7.74 | 8.23 | 8.73 | 9.19 | 9.58 | 9.80 |
| 15 |  | 6.93 | 7.38 | 8.04 | 8.70 | 9.29 | 9.78 | 10.06 |  | 7.46 | 7.76 | 8.25 | 8.74 | 9.21 | 9.60 | 9.83 |
| 16 |  | 6.89 | 7.35 | 8.04 | 8.71 | 9.31 | 9.80 | 10.07 |  | 7.48 | 7.78 | 8.25 | 8.74 | 9.21 | 9.61 | 9.85 |
| 17 |  | 6.81 | 7.30 | 8.02 | 8.70 | 9.29 | 9.78 | 10.05 |  | 7.50 | 7.78 | 8.25 | 8.74 | 9.21 | 9.62 | 9.86 |

FMD, Flow mediated dilation.

**Supplementary Table 5:** Linear regression analysis of variables on FMD adjusted for age, gender, BMI z-score and baseline artery diameter, as well as multivariate linear regression analysis of significant variables on FMD

|  |  |  |  | Adjusted for Age, Gender, BMI z-score, Baseline artery diameter | | | | |  | Multivariate | | | | |
| --- | --- | --- | --- | --- | --- | --- | --- | --- | --- | --- | --- | --- | --- | --- |
| Category | Increment^c^ | Std Dev | | Beta^b^ | Lower CI | Upper CI |  | *p* |  | Beta^b^ | Lower CI | Upper CI |  | *p* |
| Age, yr | +1 Std Dev | 2.71 |  | 0.142 | 0.081 | 0.203 |  | <0.001^a^ |  | 0.112 | 0.043 | 0.181 |  | 0.001^a^ |
| Gender | Male (vs Female) | |  | -0.010 | -0.116 | 0.095 |  | 0.848 |  |  |  |  |  |  |
| BMI-z score | +1 Std Dev | 0.94 |  | 0.008 | -0.046 | 0.062 |  | 0.767 |  |  |  |  |  |  |
| Baseline artery diameter, mm | +1 Std Dev | 0.39 |  | -0.117 | -0.189 | -0.046 |  | 0.001 ^a^ |  | -0.071 | 0.141 | 0.000 |  | 0.049^a^ |
| Pubertal stage | +1 Stage | |  | 0.035 | -0.023 | 0.094 |  | 0.239 |  |  |  |  |  |  |
| Waist-to-Hip ratio | +1 Std Dev | 0.063 |  | -0.013 | -0.069 | 0.043 |  | 0.642 |  |  |  |  |  |  |
| Gestation | Preterm (vs Term) | |  | -0.081 | -0.266 | 0.104 |  | 0.391 |  |  |  |  |  |  |
| Birth weight (kg) | +1 Std Dev | 1.11 |  | 0.030 | -0.032 | 0.091 |  | 0.340 |  |  |  |  |  |  |
| SBP, mmHg | +1 Std Dev | 10.75 |  | 0.011 | -0.045 | 0.068 |  | 0.691 |  |  |  |  |  |  |
| DBP, mmHg | +1 Std Dev | 7.82 |  | -0.053 | -0.105 | -0.001 |  | 0.047^a^ |  | -0.061 | -0.122 | -0.001 |  | 0.048^a^ |
| Heart rate, bpm | +1 Std Dev | 13.11 |  | -0.052 | -0.112 | 0.009 |  | 0.093 |  |  |  |  |  |  |
| Glucose, mmol/L | +1 Std Dev | 0.43 |  | -0.091 | -0.153 | -0.030 |  | 0.004^a^ |  | -0.068 | -0.129 | -0.007 |  | 0.028^a^ |
| Triglyceride, mmol/L | +1 Std Dev | 0.41 |  | -0.138 | -0.198 | -0.078 |  | <0.001^a^ |  | -0.091 | -0.155 | -0.027 |  | 0.005^a^ |
| Cholesterol, mmol/L | +1 Std Dev | 0.73 |  | -0.008 | -0.069 | 0.052 |  | 0.786 |  |  |  |  |  |  |
| LDL, mmol/L | +1 Std Dev | 0.63 |  | -0.025 | -0.085 | 0.035 |  | 0.407 |  |  |  |  |  |  |
| HDL, mmol/L | +1 Std Dev | 0.37 |  | 0.103 | 0.041 | 0.165 |  | 0.001^a^ |  | 0.056 | -0.009 | 0.122 |  | 0.093 |
| Physical activity^d^ | +1 Stage | |  | 0.049 | -0.021 | 0.120 |  | 0.171 |  |  |  |  |  |  |

BMI z-score: Body mass index z-score; CI: Confidence interval; DBP: Diastolic blood pressure; FMD: Flow mediated dilation; HDL: High-density lipoprotein; LDL: Low-density lipoprotein; SBP: Systolic blood pressure; Std dev: Standard deviation.
^a^ Denotes statistical significance at 0.05.
^b^ Beta for all categorical variables including gender, pubertal stage, gestation and physical activity are reported as unstandardised beta with displayed change as increment. Beta for all continuous variables are reported as standardised beta with respective standard deviation as increment.

^c^ Increment / Change for all categorical variables are reported as displayed, and for all continuous variables are reported as one additional standard deviation

^d^ Physical activity is graded in three stages – mild, moderate and vigorous.

**Supplementary Table 6:** Adjusted flow mediated dilatation (FMD) by age and gender for current study vs. Hopkins et al.^8^

| Age, Yr |  | Male | | | | |  | Female | | | | |
| --- | --- | --- | --- | --- | --- | --- | --- | --- | --- | --- | --- | --- |
|  |  | Current Study | |  | Hopkins et al^8^ | |  | Current Study | |  | Hopkins et al^8^ | |
|  |  | *n* | Adjusted FMD, % |  | *n* | Adjusted FMD, % |  | *n* | Adjusted FMD, % |  | *n* | Adjusted FMD, % |
| 8 |  | 57 | 8.23 (7.99 - 8.47) |  | 29 | 10.23 (7.66 - 12.85) | | 57 | 8.16 (7.91 - 8.40) |  | 11 | 13.89 (9.70 - 18.25) |
| 9 |  | 56 | 8.59 (8.36 - 8.83) |  | 43 | 8.03 (6.00 - 10.11) |  | 56 | 8.51 (8.27 - 8.76) |  | 23 | 9.30 (6.43 - 12.24) |
| 10 |  | 56 | 8.38 (8.14 - 8.61) |  | 65 | 9.56 (7.88 - 11.27) |  | 54 | 8.33 (8.08 - 8.57) |  | 68 | 9.57 (7.89 - 11.28) |
| 11 |  | 57 | 8.49 (8.26 - 8.72) |  | 78 | 8.31 (6.77 - 9.87) |  | 52 | 8.27 (8.02 - 8.51) |  | 72 | 9.25 (7.65 - 10.86) |
| 12 |  | 92 | 8.32 (8.13 - 8.50) |  | 53 | 8.13 (6.24 - 10.06) |  | 90 | 8.57 (8.39 - 8.76) |  | 53 | 7.79 (5.96 - 9.65) |
| 13 |  | 99 | 8.61 (8.43 - 8.79) |  | 40 | 8.10 (5.93 - 10.30) |  | 96 | 8.77 (8.59 - 8.95) |  | 51 | 8.52 (6.61 - 10.47) |
| 14 |  | 99 | 8.86 (8.67 - 9.04) |  | 46 | 7.98 (4.67 - 8.93) |  | 94 | 8.71 (8.53 - 8.89) |  | 33 | 6.25 (3.90 - 8.65) |
| 15 |  | 86 | 8.73 (8.54 - 8.93) |  | 42 | 6.78 (6.22 - 20.35) |  | 79 | 8.65 (8.45 - 8.84) |  | 35 | 7.84 (5.49 - 10.24) |
| 16 |  | 86 | 8.76 (8.56 - 8.96) |  | 34 | 7.05 (4.76 - 9.40) |  | 87 | 8.63 (8.44 - 8.82) |  | 36 | 9.43 (7.12 - 11.80) |
| 17 |  | 71 | 8.52 (8.30 - 8.74) |  | 30 | 5.98 (3.56 - 8.46) |  | 74 | 8.67 (8.46 - 8.87) |  | 21 | 8.55 (5.41 - 11.79) |

Data presented as Mean (95% confidence interval of mean) for adjusted FMD.
FMD: Flow mediated dilation.

**Supplementary Figure 1**: Bland Altman plot of subgroup of patients with paired FMD measurements at baseline and within 4 weeks later


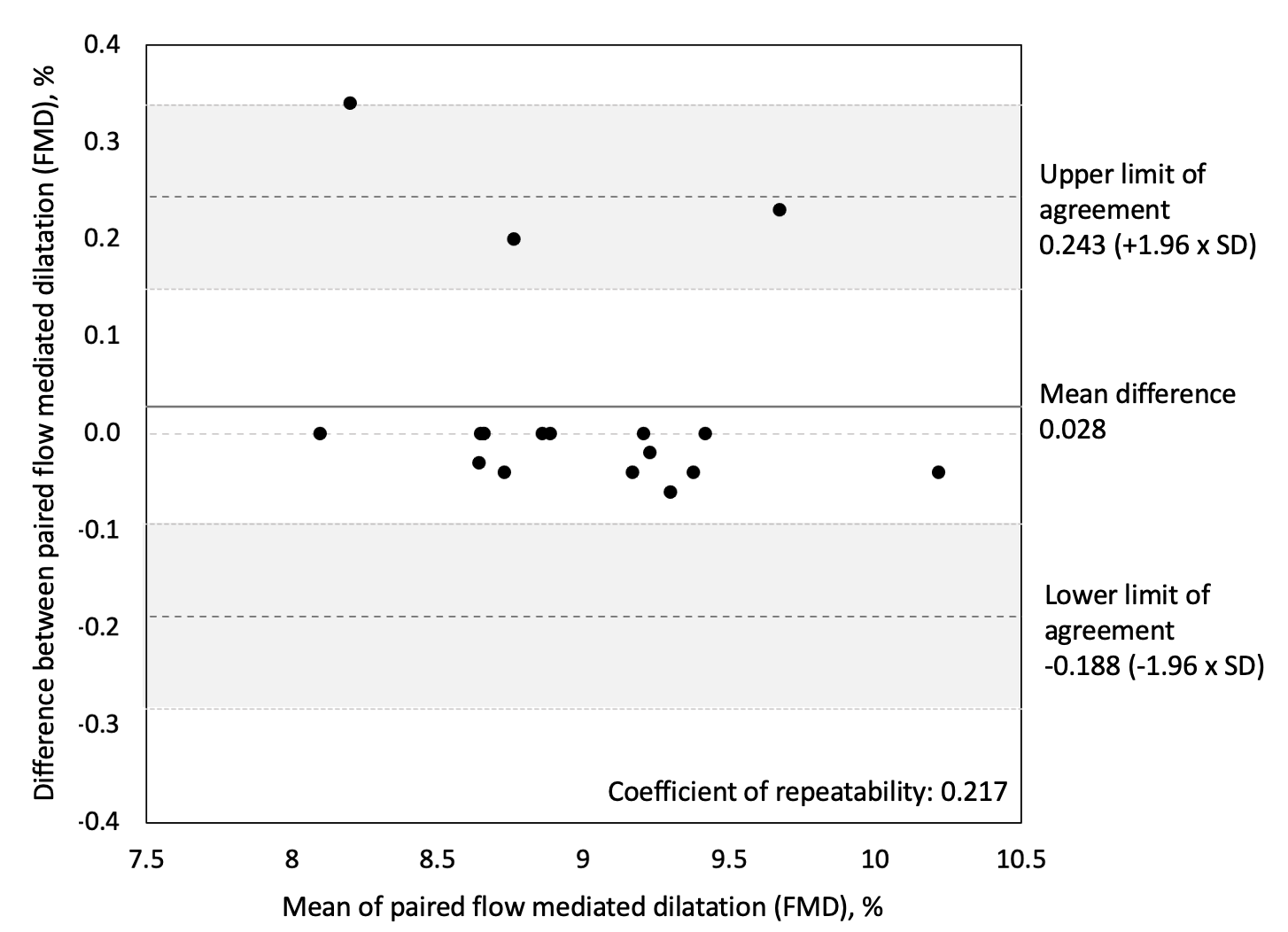


Paired measurements are represented by black circular markers.
Limits of agreement (LoA) are calculated as the mean of differences between two measurements ± 1.96 of their standard deviation.
Shaded areas represent the 95% confidence interval of the limit of agreements.

SD: Standard deviation.
